# Roxadustat Inhibits Osteoclast Differentiation and Function by Disrupting Cell Cycle Exit

**DOI:** 10.3390/ijms27125506

**Published:** 2026-06-18

**Authors:** Afang Li, Li Zuo, Luyao Li, Liangying Gan, Mi Wang, Yaoxian Liang, Qicheng Li, Xinju Zhao

**Affiliations:** 1 Department of Nephrology, Peking University People’s Hospital, Beijing 100044, China; dreamli18@126.com (A.L.); zuoli@bjmu.edu.cn (L.Z.); liluyaojd@163.com (L.L.); ganl@bkmu.edu.cn (L.G.); miqueen@163.com (M.W.); liangyaoxian2010@163.com (Y.L.); 2 Department of Trauma and Orthopedics, Peking University People’s Hospital, Beijing 100044, China

**Keywords:** roxadustat, hypoxia-inducible factor, cell cycle, osteoclast differentiation, bone resorption

## Abstract

Bone remodeling relies on a balance between osteoclast-mediated resorption and osteoblast-mediated formation. Roxadustat, a hypoxia-inducible factor prolyl hydroxylase inhibitor, promotes osteoblast differentiation but its effects on osteoclasts remain unclear. This study investigated roxadustat’s impact on osteoclast differentiation and function in vitro using primary murine bone marrow-derived mononuclear cells differentiated with M-CSF and RANKL. Cell viability, TRAP staining, bone resorption assays, RNA-seq, flow cytometry, immunofluorescence, Western blot for p27, and rescue experiments with the cyclin-dependent kinases 4 and 6 (CDK4/6) inhibitor abemaciclib were performed. Roxadustat suppressed osteoclast differentiation and resorption without cytotoxicity in a concentration-dependent manner. RNA-seq revealed enrichment of cell cycle pathways; although differentiation was inhibited, roxadustat paradoxically promoted osteoclast precursor proliferation, evidenced by increased Ki67 and decreased p27 expression. The inhibitory effects on osteoclastogenesis and resorption were partially reversed by abemaciclib. Given that terminal differentiation typically requires cell cycle exit, these findings suggest that roxadustat may inhibit osteoclast differentiation at least in part by disrupting this process, promoting precursor proliferation, and downregulating p27. Together with its known anabolic effects on osteoblasts, roxadustat might have dual therapeutic potential for bone disorders with renal anemia, such as osteoporosis in chronic kidney disease.

## 1. Introduction

Bone is a dynamic organ that undergoes continuous remodeling, a process involving the resorption of damaged or aged bone by osteoclasts and the subsequent formation of new bone by osteoblasts. This coordinated process is regulated by a variety of cytokines, metabolic activities, and signaling pathways. Under normal physiological conditions, a delicate balance is maintained between bone formation and resorption [1,2]. However, this equilibrium can be disrupted in pathological states or due to the effects of certain medications, leading to disorders such as osteoporosis, fractures, or heterotopic ossification [3].

In most organs and tissues, the physiological oxygen partial pressure (pO_2_) typically ranges from approximately 14 to 65 mmHg (2–9%) [4]. In contrast, the bone marrow (BM) maintains a hypoxic microenvironment. Direct in vivo measurements in murine BM have revealed mean extravascular pO_2_ values between 4.8 and 21.1 mmHg (0.6–2.8%) [5], despite its high vascular density. This notable reduction in oxygen tension suggests that oxygen availability may play a critical regulatory role in bone homeostasis and remodeling. Therefore, elucidating the effects of hypoxia on bone remodeling could be of considerable importance for the treatment of bone metabolic diseases.

The hypoxia-inducible factor (HIF) is a key transcription factor that mediates cellular responses to hypoxia. It consists of a functional α subunit (HIF-1α, HIF-2α, and HIF-3α) and a constitutively expressed β subunit. Under normoxia, HIF-α subunits are rapidly hydroxylated by prolyl hydroxylases (PHDs), leading to their ubiquitination and subsequent proteasomal degradation. Under hypoxia, however, PHDs activity is reduced, resulting in the stabilization of HIF-α. The accumulated HIF-α translocates to the nucleus, dimerizes with HIF-β, and activates the transcription of downstream target genes [6,7].

Roxadustat, a novel hypoxia-inducible factor prolyl hydroxylase inhibitor (HIF-PHI), can mimic a hypoxic state by stabilizing HIF-α and has been used for the treatment of renal anemia in recent years [8]. Accumulating evidence indicates that HIF plays a significant role in regulating skeletal development and bone remodeling [9,10,11]. It is widely accepted that stabilizing HIF-1α promotes osteoblast differentiation and enhances bone formation [12,13,14]. Consistent with this, our previous study demonstrated that roxadustat promotes osteoblast differentiation and mineralization, and ameliorates osteoporosis in ovariectomized rat models through HIF-1α stabilization [15]. However, the effect of roxadustat on osteoclast differentiation remains unclear. Therefore, this study aims to investigate the effect of roxadustat on osteoclasts in vitro and to explore its underlying mechanisms using transcriptome sequencing.

## 2. Results

### 2.1. Roxadustat Inhibited RANKL-Induced Osteoclast Differentiation

**Figure 1:**
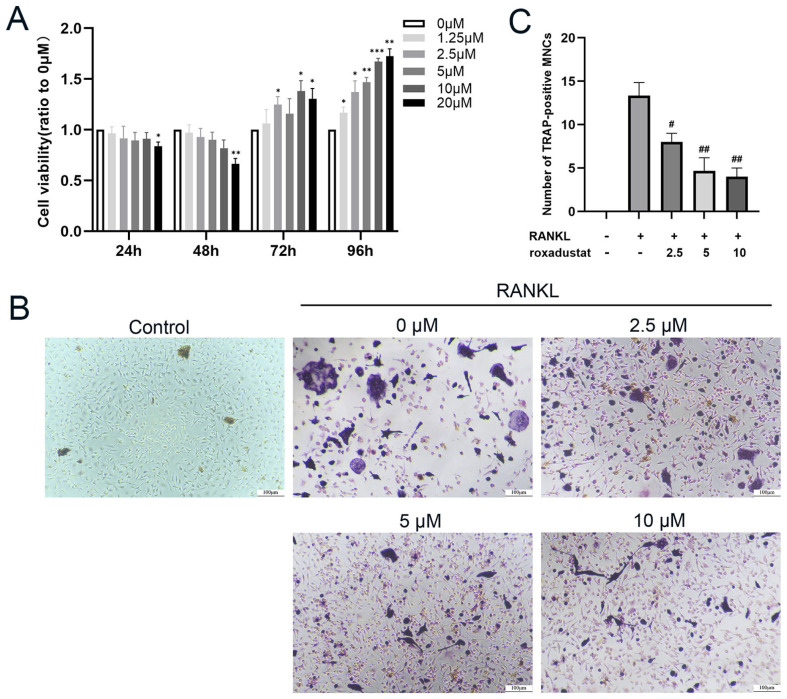
Roxadustat inhibited RANKL-induced osteoclast differentiation in a concentration-dependent manner without cytotoxicity. (**A**) The viability of osteoclast precursors treated with roxadustat at different concentrations (0–20 μM) for 24 to 96 h. (**B**) Representative images of TRAP-stained osteoclasts captured under a light microscope. Scale bars: 100 μm. (**C**) Quantitative analysis of the number of TRAP-positive multinucleated cells (MNCs; ≥3 nuclei) per field. Data are presented as the mean ± SD of three independent experiments; * *p* < 0.05, ** *p* < 0.01, *** *p* < 0.001 vs. the control group (RANKL−, roxadustat−); ^#^ *p* < 0.05, ^##^ *p* < 0.01 vs. the positive control group (RANKL+, roxadustat−).

To exclude the possibility that the effect of roxadustat on osteoclast differentiation was due to cytotoxicity, we assessed its impact on cell viability using the CCK-8 assay. The results indicated that treatment with roxadustat for 24 h did not significantly affect cell viability at concentrations ≤ 10 μM. Furthermore, prolonged exposure to roxadustat even enhanced cell proliferation (Figure 1A). Based on these findings, we selected roxadustat concentrations of 2.5, 5, and 10 μM, which exhibited minimal effects on cell viability, for subsequent experiments.

We next investigated the effect of various concentrations of roxadustat on osteoclast differentiation, as assessed by the formation of TRAP-positive multinucleated cells (MNCs; ≥3 nuclei), a hallmark of mature osteoclasts. RANKL stimulation successfully induced osteoclast differentiation and served as the positive control group (Figure 1B). However, the addition of roxadustat significantly reduced the number of osteoclasts in a concentration-dependent manner (Figure 1B,C).

### 2.2. Roxadustat Inhibited Osteoclast Bone Resorption and Altered Osteoclast-Related Gene Expression

**Figure 2:**
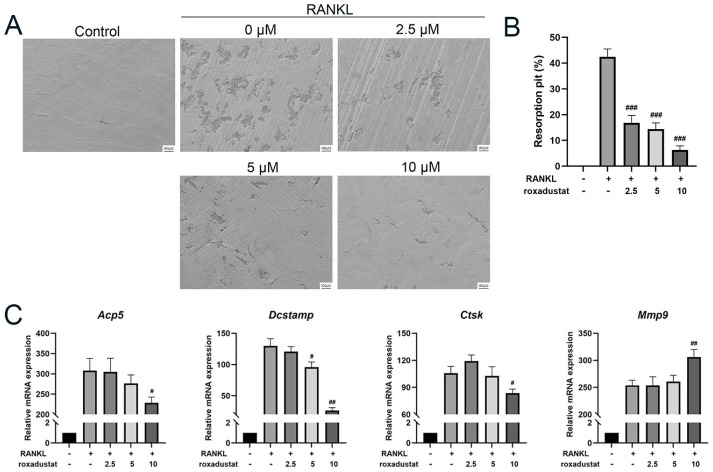
Roxadustat suppressed bone resorption function of osteoclasts and altered the expression of differentiation-related genes. (**A**) Representative images of bone resorption pit captured under a scanning electron microscopy. Scale bars: 100 μm. (**B**) Quantitative analysis of the percentage of resorption pit area relative to the total area per field. (**C**) The mRNA relative expression level of *Acp5*, *Dcstamp*, *Ctsk* and *Mmp9* normalized to *Actb*. Data are presented as the mean ± SD of three independent experiments; ^#^ *p* < 0.05, ^##^ *p* < 0.01, ^###^ *p* < 0.001 vs. the positive control group (RANKL+, roxadustat−).

Bone resorption is a critical function of mature osteoclasts. In this study, osteoclast precursors were first induced for osteoclast differentiation over 5 days, followed by an additional 3 days of maintenance culture to allow complete bone resorption. The bone slices were then examined by scanning electron microscopy to assess resorption pits. The positive control group exhibited extensive bone resorption areas (Figure 2A). In contrast, the addition of roxadustat markedly reduced the bone resorption area in a concentration-dependent manner, with the 10 μM roxadustat-treated group showing the smallest resorption pit area (Figure 2A,B).

We further analyzed the expression of osteoclast-related genes by qPCR, assessing the mRNA levels of *Acp5*, *Dcstamp*, *Ctsk*, and *Mmp9* in each group. The results demonstrated that 10 μM roxadustat significantly suppressed the expression of *Acp5*, *Dcstamp*, and *Ctsk* compared with the positive control group (Figure 2C). Interestingly, the same concentration of roxadustat led to elevated *Mmp9* expression, whereas no significant effects were observed at other concentrations (Figure 2C).

### 2.3. Identification and Enrichment Analysis of Differentially Expressed Genes (DEGs)

**Figure 3:**
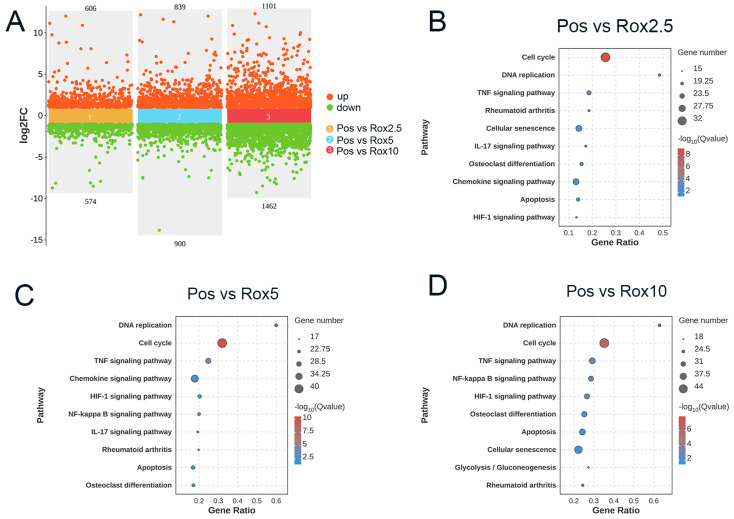
Differential expression profiling and Kyoto Encyclopedia of Genes and Genomes (KEGG) pathway enrichment analysis. (**A**) Scatter plots showing differentially expressed genes (DEGs) between the positive control group (Pos) and roxadustat-treated groups at various concentrations (2.5 μM, Rox2.5; 5 μM, Rox5; 10 μM, Rox10). Each point represents an individual gene. (**B**–**D**) KEGG pathway enrichment bubble plots for DEGs identified in the Rox2.5 (**B**), Rox5 (**C**), and Rox10 (**D**) groups compared with Pos. Bubble size corresponds to the number of DEGs enriched in the corresponding pathway. Bubble color indicates the statistical significance of enrichment, expressed as −log10 (Qvalue), where Qvalue refers to the adjusted *p*-value.

To elucidate the key signaling pathways modulated by roxadustat during osteoclast differentiation, we performed RNA-seq analysis on RANKL-induced osteoclasts treated with or without various concentrations of roxadustat. DEGs for each comparison were identified using thresholds of |log_2_(fold change)| > 1 and an adjusted *p*-value < 0.05. Scatter plots were generated to visualize transcriptional differences between the positive control group (Pos) and roxadustat-treated groups (2.5 μM, Rox2.5; 5 μM, Rox5; 10 μM, Rox10) (Figure 3A). The 10 μM roxadustat group (Rox10) showed the most transcriptional changes, with 1101 upregulated and 1462 downregulated genes, indicating a concentration-dependent effect on the transcriptome.

Kyoto Encyclopedia of Genes and Genomes (KEGG) pathway enrichment analysis of DEGs from each comparison revealed several significantly enriched biological pathways. Compared with Pos, roxadustat treatment led to significant enrichment of pathways related to the cell cycle, DNA replication, osteoclast differentiation, HIF-1 signaling, among others (Figure 3B–D). Notably, the cell cycle pathway was the most significantly enriched, suggesting that roxadustat may play an important role in regulating cellular proliferation and division.

Based on these transcriptional changes and prior observations, we hypothesized that roxadustat modulates osteoclast differentiation through the regulation of “cell cycle”. The 10 μM concentration was selected for subsequent experiments.

### 2.4. Roxadustat Promoted Cell Cycle Progression of Osteoclast Precursors

**Figure 4:**
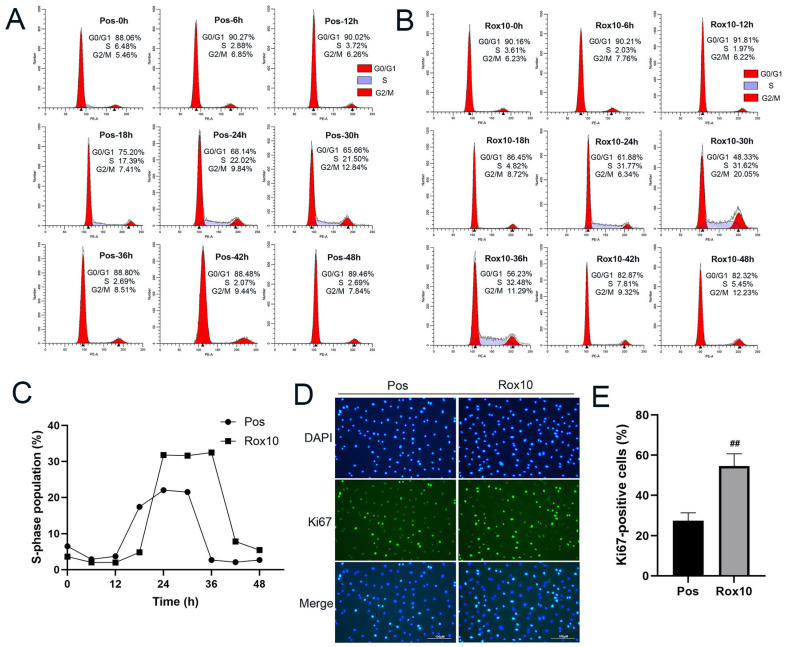
Roxadustat promoted cell cycle progression and proliferation of osteoclast precursors. (**A**,**B**) Flow cytometry analysis of cell cycle distribution in osteoclast precursors treated without (Pos, (**A**)) or with (Rox10, (**B**)) 10 μM roxadustat for the indicated times. Percentages of cells in each phase (G0/G1, S, and G2/M) are indicated. (**C**) Dynamic proportion of S-phase cells in Pos and Rox10 groups measured at 6 h intervals over a 48 h period. (**D**) Representative immunofluorescence images of Ki67 staining (green) at 48 h. Nuclei were counterstained with DAPI (blue). Scale bars: 100 μm. (**E**) Quantitative analysis of the percentage of Ki67-positive cells. Data are presented as the mean ± SD of three independent experiments; ^##^ *p* < 0.01 vs. the Pos group.

To investigate alterations in the cell cycle during osteoclast differentiation, we analyzed the cell cycle distribution of osteoclast precursors over 48 h in the presence or absence of 10 μM roxadustat using flow cytometry (Figure 4A,B). The proportion of cells in S phase at 6 h intervals is shown in Figure 4C. In the Pos group, the S-phase population increased significantly at 18 h, followed by a marked decline at 36 h. In contrast, the Rox10 group exhibited delayed entry into S phase but reached a higher peak proportion. Furthermore, immunofluorescence staining for Ki67 at 48 h revealed a significantly higher percentage of Ki67-positive cells in the Rox10 group compared with the Pos group (Figure 4D,E), indicating that roxadustat promotes the proliferation of osteoclast precursors.

### 2.5. Cell Cycle Arrest Alleviated the Inhibition of Osteoclast Differentiation Caused by Roxadustat

To investigate whether inhibiting the rapid proliferation of osteoclast precursors could alleviate the suppressive effect of roxadustat on osteoclast differentiation, we added abemaciclib (Abe, 1 μM), a selective and potent inhibitor of cyclin-dependent kinases 4 and 6 (CDK4/6) that induces cell cycle arrest by blocking the G1-to-S phase transition [16], into the osteoclastogenic induction medium containing 10 μM roxadustat.

**Figure 5:**
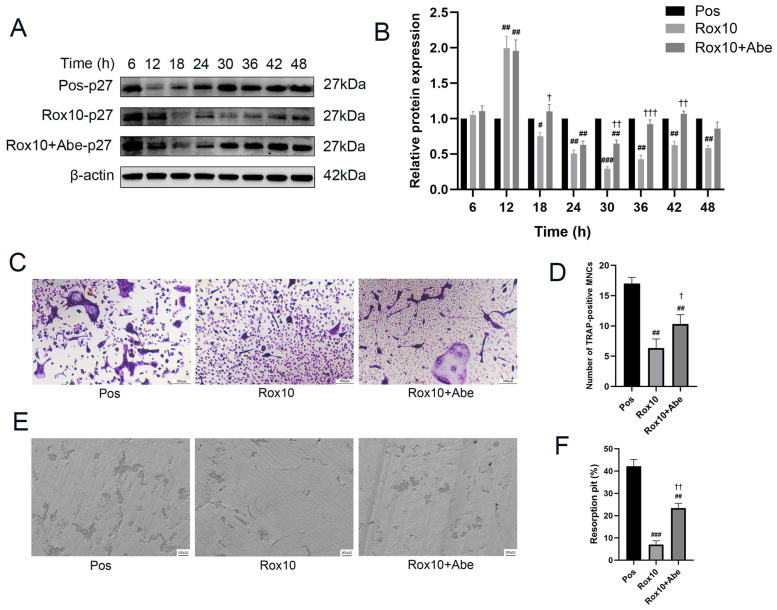
Abemaciclib alleviated roxadustat-treated suppression of osteoclastogenesis by modulating cell cycle progression. (**A**) Western blot analysis of p27 protein expression at the indicated time points in the positive control (Pos), 10 μM roxadustat (Rox10), and Rox10 + abemaciclib (Rox10 + Abe) groups. (**B**) Densitometric quantification of osteoclast precursors p27 protein levels normalized to β-actin. (**C**) Representative images of TRAP-stained osteoclasts captured under a light microscope. Scale bars: 100 μm. (**D**) Quantitative analysis of the number of TRAP-positive MNCs per field. (**E**) Representative images of bone resorption pit captured under a scanning electron microscopy. Scale bars: 100 μm. (**F**) Quantitative analysis of the percentage of resorption pit area relative to the total area per field. Data are presented as the mean ± SD of three independent experiments; ^#^ *p* < 0.05, ^##^ *p* < 0.01, ^###^ *p* < 0.001 vs. the Pos group (RANKL+, roxadustat−); ^†^ *p* < 0.05, ^††^ *p* < 0.01, ^†††^ *p* < 0.001 vs. the Rox10 group (RANKL+, 10 μM roxadustat+).

We first examined the expression of p27, a critical cell cycle inhibitory protein. In the Pos group, p27 expression remained relatively stable throughout the time course. In contrast, the Rox10 group showed a significant decline in p27 levels, particularly after 18 h. The addition of abemaciclib increased p27 levels compared with the Rox10 group, and no significant difference was observed relative to the Pos group after 36 h (Figure 5A,B). Concurrently, we assessed the impact of abemaciclib on cell cycle distribution. Flow cytometry analysis revealed that abemaciclib treatment induced significant G0/G1 phase arrest accompanied by a reduction in the S-phase population, both with or without roxadustat (Figure S1). These results indicate that abemaciclib successfully induced cell cycle arrest.

We next evaluated osteoclast formation and function using TRAP staining and bone resorption pit assays. The addition of abemaciclib significantly alleviated roxadustat-treated inhibition of both osteoclast formation and resorption function, although the values remained lower than those in the Pos group (Figure 5C–F). We further examined whether abemaciclib alone influenced osteoclast differentiation and resorption. Notably, abemaciclib treatment alone did not affect the number of mature osteoclasts but impaired their resorption function compared with the Pos group (Figure S2).

## 3. Discussion

Osteoclasts, as terminally differentiated cells derived from the monocyte/macrophage lineage, play an essential role in bone resorption, a critical process for bone remodeling and calcium homeostasis [17]. The role of HIF signaling in osteoclastogenesis remains controversial, with reports suggesting both pro- and anti-osteoclastogenic effects depending on the context, model, and specific HIF-α isoform involved [18,19,20]. In this study, we found that the pan-HIF stabilizer roxadustat inhibited RANKL-induced osteoclast differentiation and suppressed the resorption function of mature osteoclasts in vitro. Consistent with our results, other prolyl hydroxylase inhibitors such as dimethyloxalylglycine (DMOG) and deferoxamine (DFO) have also been shown to suppress osteoclastogenesis [21]. Moreover, the downregulation of key osteoclast genes including *Acp5*, *Ctsk*, and *Dcstamp* by roxadustat treatment provided a molecular basis for the impaired differentiation and function. However, the expression of *Mmp9* was elevated in the 10 μM roxadustat-treated group. This discrepancy may be attributed to the complex multi-factorial regulation of *Mmp9*, which has been previously reported to be promoted by HIF-1α [22,23].

Transcriptomic analysis suggested that the cell cycle pathway may be among the most significantly affected by roxadustat. Subsequent cell cycle analysis indicated that while roxadustat delayed the S-phase entry of osteoclast precursors, it ultimately amplified proliferation. This was further supported by immunofluorescence staining showing a higher percentage of Ki67-positive cells at 48 h, suggesting that roxadustat pushes precursors into a heightened state of proliferation. However, terminal cell differentiation is typically coupled with cell cycle exit, which is a well-established paradigm in cellular biology. For instance, in adipocytes, terminal differentiation occurs through a competition during a lengthening G1 phase [24], and reduced proliferation of muscle stem cells facilitates myoblast differentiation [25]. In line with this phenomenon, our results showed that although roxadustat enhanced the proliferation of osteoclast precursors, it ultimately reduced the number of mature osteoclasts.

The rescue experiment using abemaciclib, a CDK4/6 inhibitor, strongly supports this mechanistic model. By inducing G0/G1 arrest and restoring p27 expression, abemaciclib significantly mitigated the inhibitory effects of roxadustat on both osteoclast formation and resorption. Accumulating evidence highlights that cell cycle arrest is an indispensable prerequisite for initiating terminal osteoclast differentiation. In addition to the well-established role of p27 [26,27], p15 has recently been identified as a novel critical regulator of osteoclast differentiation. Stage-specific upregulation of p15 and subsequent induction of G0/G1 phase arrest are indispensable for osteoclast differentiation, as evidenced by impaired formation of TRAP-positive multinucleated osteoclasts and downregulation of differentiation-related genes upon its silencing [28]. Furthermore, synchronized G0/G1 phase arrest, induced by transient M-CSF deprivation, potently enhances osteoclast differentiation of murine bone marrow-derived progenitors [29]. Interestingly, our results showed that abemaciclib alone did not promote differentiation and even impaired resorption function. We speculate that this may be attributed to the pharmacological properties of abemaciclib, particularly its pro-apoptotic effects [30,31], which may compromise osteoclast function.

Osteoclast differentiation is a highly energy-demanding process, and emerging evidence indicates its heavily reliant on oxidative phosphorylation (OXPHOS) to meet its substantial bioenergetic and biosynthetic requirements [32,33]. In contrast, metabolic reprogramming from OXPHOS toward glycolysis has been shown to inhibit osteoclast formation [34]. The highly proliferative state induced by roxadustat is likely associated with a metabolic switch toward glycolysis. This aligns with the established role of HIF-1α, which promotes glycolytic metabolism and cell cycle progression under hypoxic conditions to support bioenergetic and proliferative demands [35]. Therefore, it remains to be determined whether impaired differentiation results directly from dysregulated cell cycle control, from inadequate metabolic adaptation that compromises fusion and resorption function, or from a combination of both mechanisms. This represents an important direction for future research.

Roxadustat exhibits dual effects on bone metabolism by promoting osteoblast differentiation while inhibiting osteoclast differentiation [15,36], thereby demonstrating therapeutic potential for bone-related disorders. However, its potential impact on fracture healing warrants careful consideration, as roxadustat-induced suppression of osteoclast activity may theoretically delay bone remodeling. Meanwhile, fracture itself promotes osteoclast formation through the release of damage-associated molecular patterns (e.g., SAP-130) from necrotic osteocytes, which activate the Mincle receptor on osteoclast precursors and enhance bone resorption [37]. These opposing effects are likely to interact in a complex manner, and the net outcome of roxadustat on fracture repair may depend on factors such as timing, dosage, and the local microenvironment. In addition, systemic administration of roxadustat for the treatment of renal anemia has been associated with adverse effects, such as thromboembolic events [38,39]. Therefore, local delivery strategies, such as incorporating roxadustat into biocompatible scaffolds, hydrogels, or bone cements, offer a promising approach to achieve high drug concentrations at the fracture site while minimizing systemic exposure. Consequently, further studies are warranted to evaluate the efficacy and safety of locally applied roxadustat in animal models of fracture or bone defect.

There are several limitations to our study. First, while the use of an in vitro system is common in this field, it may not fully replicate the complexity of the in vivo bone marrow microenvironment, where HIF signaling occurs within a multicellular network involving osteoblasts, immune cells, and vascular endothelial cells. Therefore, although our results indicate that roxadustat inhibits osteoclast formation in vitro, the physiological relevance would benefit from validation in more advanced models, such as co-culture systems or animal studies; in addition, prospective clinical follow-up studies in patients receiving roxadustat for renal anemia could provide valuable insight into its in vivo effects on bone remodeling, especially on osteoclast-mediated resorption. Second, the precise mechanism by which roxadustat influences the cell cycle during osteoclast differentiation has not been fully elucidated. Although changes in p27 expression were observed following treatment, the underlying molecular mechanisms, including potential modulation by HIF or interactions with other cyclin/CDK complexes, remain to be characterized. Third, while our study focused on p27 and cell cycle distribution, other cell cycle regulators such as p15 and p21 were not examined. Further studies incorporating a broader panel of markers and investigating post-translational modifications are warranted.

## 4. Materials and Methods

### 4.1. Cell Isolation and Culture

Bone marrow-derived mononuclear cells were isolated from 6-week-old wild-type male C57BL/6J mice as reported previously [40]. The mice were purchased from Beijing Vital River Laboratory Animal Technology Co., Ltd. (Beijing, China), and were housed under specific pathogen-free conditions. Briefly, mice were euthanized by CO_2_ inhalation followed by cervical dislocation, in accordance with the AVMA Guidelines and standard practice for rodents. Bone marrow was immediately flushed from the tibias and femurs with cold phosphate-buffered saline (PBS). Erythrocytes were removed by treatment with an erythrocyte lysis buffer (Solarbio, Beijing, China), and the remaining cells were cultured for 24 h in complete medium, α-Minimum Essential Medium (α-MEM; Gibco, Waltham, MA, USA) supplemented with 10% fetal bovine serum (FBS; Gibco, Waltham, MA, USA), 100 U/mL penicillin and 100 µg/mL streptomycin (Gibco, Waltham, MA, USA). Non-adherent cells were collected and then cultured in complete medium supplemented with 30 ng/mL M-CSF (PeproTech, Cranbury, NJ, USA) for subsequent experiments. After M-CSF culture, the adherent cells were used as osteoclast precursors. A total of 30 mice were used as the source of bone marrow-derived mononuclear cells, which were obtained from 6 independent isolations. For each isolation, cells from 5 mice were pooled and subsequently cultured, and the comparisons were performed at the cellular level. All animal experiments were conducted in compliance with the Guide for the Care and Use of Laboratory Animals issued by the US National Institute of Health (NIH Publication No. 85-23). All animal procedures were approved by the Institutional Authority for Laboratory Animal Care of Peking University People’s Hospital (approval number: 2022PHE042, approval date: 22 July 2022).

### 4.2. Cell Viability Assay

Cell viability was assessed using the cell counting kit-8 (CCK-8; Dojindo, Kumamoto, Japan) according to the manufacturer’s instructions. Briefly, cells were seeded in 96-well plates at a density of 5 × 10^3^ cells per well in 100 µL of complete medium supplemented with 30 ng/mL M-CSF and allowed to adhere overnight. Then roxadustat (FG-4592; MedChemExpress, Monmouth Junction, NJ, USA) was added to the medium at various concentrations (0 μM, 1.25 µM, 2.5 µM, 5 µM, 10 µM, 20 µM). After treatment with roxadustat for 24, 48, 72, and 96 h, the culture medium was carefully removed and replaced with 100 µL of fresh medium containing 10% CCK-8 reagent (*v*/*v*). Plates were incubated at 37 °C for 2 h. The absorbance of each well was measured at a wavelength of 450 nm using a microplate reader (Bio-Rad, Hercules, CA, USA).

### 4.3. Osteoclast Differentiation and TRAP Staining

Osteoclast precursors were cultured in complete medium supplemented with 30 ng/mL M-CSF until reaching 60–80% confluence. Then the medium was replaced with an osteoclastogenic induction medium containing 50 ng/mL RANKL (R&D Systems, Minneapolis, MN, USA) and various concentrations of roxadustat to induce osteoclast differentiation. After 5 days, the cells were stained for tartrate resistant acid phosphatase (TRAP) using a commercial kit (PH Biotechnology, Wuxi, China) according to the manufacturer’s instructions. TRAP-positive multinucleated cells (MNCs; ≥3 nuclei) were identified and counted as mature osteoclasts under a light microscope (Nikon, Tokyo, Japan).

### 4.4. Real-Time Quantitative PCR (qPCR)

Osteoclast precursors were seeded at a density of 1 × 10^5^ cells per well in 12-well plates and cultured in osteoclastogenic induction medium with or without roxadustat for 5 days, then total RNA was extracted from cultured cells using TRIzol kit (Beyotime, Shanghai, China). 500 ng of total RNA from each sample was reverse-transcribed into cDNA using PrimeScript RT reagent Kit (Takara, Kusatsu, Shiga, Japan). For qPCR, samples were performed using TB Green Premix Ex Taq II (Takara, Kusatsu, Shiga, Japan) with the CFX96 Real-Time PCR Detection System (Bio-Rad, Hercules, CA, USA). The primers used are summarized in Table S1, the abundance of each target mRNA was normalized by that of *Actb* mRNA.

### 4.5. Bone Resorption Pit Assay

The resorption function of mature osteoclasts was evaluated using a bone slice assay. Briefly, osteoclast precursors were seeded on bovine cortical bone slices (IDS, Boldon, UK) in 96-well plates, following a 5-day osteoclast induction period and 3-day bone resorption period, the cellular layers were thoroughly removed from the bone slices by ultrasonication. Bone slices were then rinsed with distilled water, air-dried and examined using a scanning electron microscope (JEOL, Tokyo, Japan). The area of resorption pits was quantified using ImageJ software (version 1.54; National Institutes of Health, Bethesda, MD, USA).

### 4.6. RNA-seq and Bioinformatics Analysis

Following a 5-day induction of osteoclast differentiation from osteoclast precursors exposed to various concentrations of roxadustat, with three replicates respectively, total RNA was extracted from the induced osteoclasts. RNA integrity was assessed using the RNA Nano 6000 Assay Kit of the Agilent Bioanalyzer 2100 system (Agilent Technologies, Santa Clara, CA, USA). A total amount of 3 µg RNA per sample was used as input material for the RNA sample preparations. Sequencing libraries were generated using NEB Next^®^ Ultra™ RNA Library Prep Kit for Illumina^®^ (NEB, Ipswich, MA, USA) following manufacturer’s recommendations and index codes were added to attribute sequences to each sample. The clustering of the index-coded samples was performed on a cBot Cluster Generation System using TruSeq PE Cluster Kit v3-cBot-HS (Illumina, San Diego, CA, USA) according to the manufacturer’s instructions. After cluster generation, the library preparations were sequenced on an Illumina Nova seq6000 platform and 150 bp paired-end reads were generated. Bioinformatics analysis for DEGs was performed using the Omicsmart tools at www.omicsmart.com.

### 4.7. Cell Cycle Analysis

For cell cycle analysis, osteoclast precursors were seeded at a density of 1 × 10^5^ cells per well in 12-well plates. Upon reaching 60–80% confluence, the cells were incubated in serum-free medium for 24 h. After synchronization, the medium was replaced with an osteoclastogenic induction medium. Following incubation, cells were collected at designated time points. Cell cycle analysis was performed by flow cytometry (BD, Franklin Lakes, NJ, USA) using propidium iodide (PI) staining (MedChemExpress, Monmouth Junction, NJ, USA) according to the manufacturer’s instructions, and the percentages of cells in G0/G1, S, and G2/M phases were calculated with ModFit LT 5.0 software (Verity Software House, Topsham, ME, USA). 

### 4.8. Immunofluorescence Staining

To assess differences in cell proliferation, immunofluorescence staining for the Ki67 protein was performed on cells. Briefly, samples were fixed with 4% paraformaldehyde for 15 min at room temperature (RT) and permeabilized with 0.1% Triton X-100 for 10 min. After blocking with 5% bovine serum albumin (BSA) in PBS for 1 h, the cells were incubated overnight at 4 °C with a primary antibody against Ki67 (Cell Signaling Technology, Danvers, MA, USA, 9129T, 1:500). The following day, after thorough washing with PBS, samples were incubated with Alexa Fluor 488-conjugated goat anti-rabbit antibody (Abcam, Cambridge, UK, ab150077, 1:1000) for 1 h at RT in the dark. Nuclei were stained with DAPI for 5 min. Images were acquired with a fluorescence microscope, and Ki67-positive cells were quantified using ImageJ software.

### 4.9. Western Blot Analysis

For analysis of cell cycle key regulators protein p27, cells were lysed by RIPA buffer (Beyotime, China) supplemented with protease inhibitor (Thermo Fisher Scientific, Waltham, MA, USA). The protein concentration was determined using a bicinchoninic acid (BCA) assay kit (Applygen, Beijing, China). Equal amounts of proteins from each sample were separated by sodium dodecyl sulfate-polyacrylamide gel electrophoresis (SDS-PAGE) on 10% gels and then transferred to polyvinylidene fluoride (PVDF) membranes (Millipore, Burlington, MA, USA). The membranes were blocked with 5% non-fat milk for 1 h at room temperature, and subsequently incubated overnight at 4 °C with primary antibody against p27 (Cell Signaling Technology, Danvers, MA, USA, 2552T, 1:1000) and β-actin (Beyotime, Shanghai, China, AF5003, 1:1000). After washing with TBST, the membranes were incubated with appropriate HRP-conjugated secondary antibody for 1 h at RT. Protein bands were visualized using an enhanced chemiluminescence (ECL) substrate (Thermo Fisher Scientific, Waltham, MA, USA) and captured with a chemiluminescence detection system (Bio-Rad, Hercules, CA, USA). The band intensities were quantified using ImageJ software, relative protein expression was normalized with β-actin.

### 4.10. Rescue Experiment

To further validate that cell cycle arrest mediates the reversal of roxadustat-induced inhibition of osteoclast differentiation and function, 1 μM abemaciclib (MedChemExpress, Monmouth Junction, NJ, USA) was used for rescue experiments. Osteoclast precursors were cultured in osteoclastogenic induction medium supplemented with or without 10 μM roxadustat, and in the presence or absence of 1 μM abemaciclib. For TRAP staining, cells were incubated for 5 days. For the resorption pit assay, cells were cultured on bone slices for 5 days, followed by an additional 3 days. For Western blot analysis of p27, cells were harvested at the indicated time points (6, 12, 18, 24, 30, 36, 42, and 48 h) after the start of induction.

### 4.11. Statistical Analysis

Data are presented as mean ± standard deviation (SD) from at least three independent experiments. Statistical analysis was performed with GraphPad Prism (version 10.6.0; GraphPad Software, San Diego, CA, USA). Differences between two groups were analyzed with Student’s *t* test, and one-way analysis of variance (ANOVA) followed by Tukey’s post hoc test was used for comparisons among three or more groups. Values were considered statistically significant at *p* < 0.05.

## 5. Conclusions

This study indicates that roxadustat inhibits osteoclast differentiation through a mechanism involving the disruption of cell cycle regulation. Specifically, roxadustat promotes the proliferation of osteoclast precursors and downregulates the cell cycle inhibitor p27, thereby impeding the requisite cell cycle exit and ultimately attenuating terminal osteoclast maturation and function. These results, combined with our prior finding that roxadustat enhances osteoblast differentiation [15], suggest that HIF-PHIs such as roxadustat could offer a therapeutic advantage by correcting anemia while concurrently modulating bone metabolism. This dual effect may be particularly beneficial for osteoporotic patients with renal anemia. Nevertheless, further investigation is necessary to elucidate the long-term effects on skeletal balance in vivo.

## Data Availability

The raw sequencing data generated in this study have been deposited in the NCBI Sequence Read Archive (SRA) under accession number PRJNA1355497, and are publicly available at https://www.ncbi.nlm.nih.gov/bioproject/PRJNA1355497 (accessed on 12 June 2026). Additional data or materials are available from the corresponding author upon reasonable request.

## Supplementary Materials

The following supporting information can be downloaded at: https://www.mdpi.com/article/10.3390/ijms27125506/s1.

## Abbreviations

The following abbreviations are used in this manuscript:

HIF	hypoxia-inducible factor
PHDs	prolyl hydroxylases
HIF-PHI	hypoxia-inducible factor prolyl hydroxylase inhibitor
MNCs	multinucleated cells
KEGG	Kyoto Encyclopedia of Genes and Genomes
CDK4/6	cyclin-dependent kinases 4 and 6
DEGs	differentially expressed genes
