# Supplementary material for: Roxadustat Inhibits Osteoclast Differentiation and Function by Disrupting Cell Cycle Exit
Source: Int J Mol Sci. 2026 Jun 18;27(12):5506. doi: 10.3390/ijms27125506 (PMC13299530; doi:10.3390/ijms27125506)
Supplement: Supplementary file 1 [file ijms-27-05506-s001.zip › ijms-4347955-supplementary.pdf]

Table S1. The primer sequences for real-time qPCR.

Gene	Forward(5'-3')	Reverse(5'-3')
Acp5	CACTCCCACCCTGAGATTTGT	CATCGTCTGCACGGTTCTG
Dcstamp	GGGGACTTATGTGTTTCCACG	ACAAAGCAACAGACTCCCAAAT
Ctsk	GAAGAAGACTCACCAGAAGCAG	TCCAGGTTATGGGCAGAGATT
Mmp9	CTGGACAGCCAGACACTAAAG	CTCGCGGCAAGTCTTCAGAG
Actb	GGCTGTATTCCCCTCCATCG	CCAGTTGGTAACAATGCCATGT

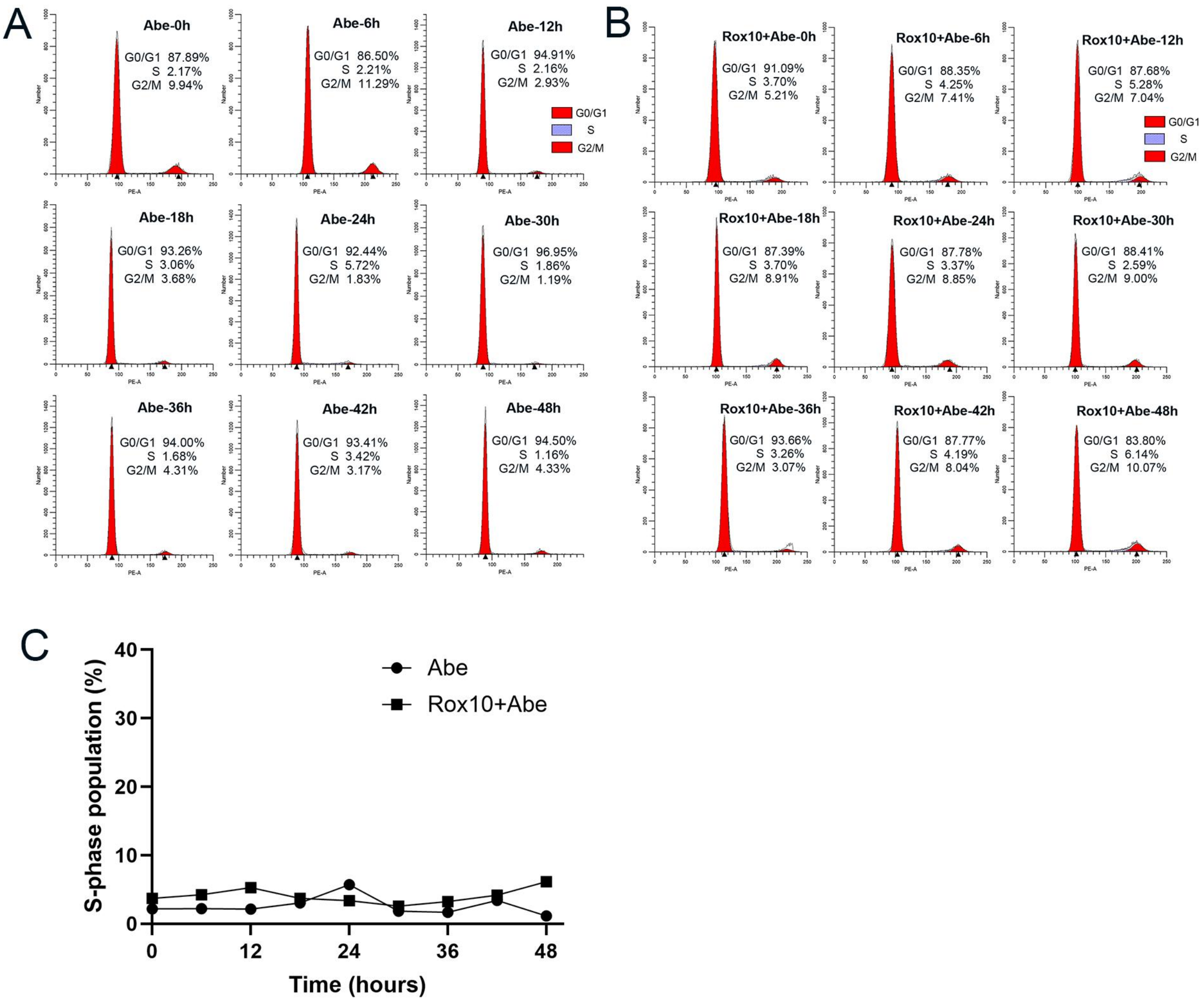

Figure S1. Abemaciclib induced G0/G1 phase arrest in osteoclast precursors. (A, B) Flow cytometry analysis of cell cycle distribution in osteoclast precursors treated with abemaciclib in the absence (Abe, A) or presence of 10 μM roxadustat (Rox10+Abe, B) for the indicated times. The percentages of cells in each phase (G0/G1, S, and G2/M) are indicated. (C) Dynamic proportion of S-phase cells in the Abe and Rox10+Abe groups measured at 6-h intervals over a 48-h period.

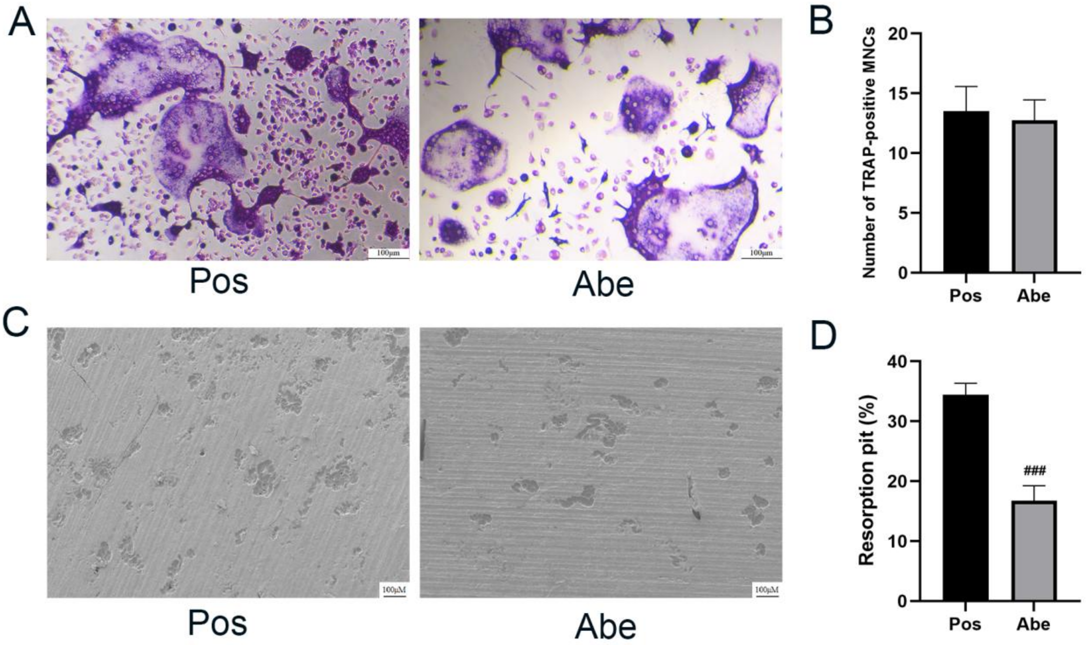

Figure S2. Abemaciclib inhibited bone resorption without affecting osteoclast differentiation. (A) Representative images of TRAP-stained osteoclasts in the positive control (Pos) and abemaciclib (Abe) groups captured under a light microscope. Scale bars: 100 μm. (B) Quantitative analysis of the number of TRAP-positive MNCs (≥3 nuclei) per field. (C) Representative images of bone resorption pit captured under a scanning electron microscopy. Scale bars: 100 μm. (D) Quantitative analysis of the percentage of resorption pit area relative to the total area per field. Data are presented as the mean ± SD of three independent experiments; ###$P < 0.001$ *vs.* the Pos group.
